# Recipes and mechanisms of cellular reprogramming: a case study on budding yeast *Saccharomyces cerevisiae*

**DOI:** 10.1186/1752-0509-5-50

**Published:** 2011-04-12

**Authors:** Shengchao Ding, Wei Wang

**Affiliations:** 1 Department of Chemistry and Biochemistry, University of California, San Diego, 9500 Gilman Drive, La Jolla, CA 92093-0359, USA

## Abstract

**Background:**

Generation of induced pluripotent stem cells (iPSCs) and converting one cell type to another (transdifferentiation) by manipulating the expression of a small number of genes highlight the progress of cellular reprogramming, which holds great promise for regenerative medicine. A key challenge is to find the recipes of perturbing genes to achieve successful reprogramming such that the reprogrammed cells function in the same way as the natural cells.

**Results:**

We present here a systems biology approach that allows systematic search for effective reprogramming recipes and monitoring the reprogramming progress to uncover the underlying mechanisms. Using budding yeast as a model system, we have curated a genetic network regulating cell cycle and sporulation. Phenotypic consequences of perturbations can be predicted from the network without any prior knowledge, which makes it possible to computationally reprogram cell fate. As the heterogeneity of natural cells is important in many biological processes, we find that the extent of this heterogeneity restored by the reprogrammed cells varies significantly upon reprogramming recipes. The heterogeneity difference between the reprogrammed and natural cells may have functional consequences.

**Conclusions:**

Our study reveals that cellular reprogramming can be achieved by many different perturbations and the reprogrammability of a cell depends on the heterogeneity of the original cell state. We provide a general framework that can help discover new recipes for cellular reprogramming in human.

## Background

In response to environmental or developmental signals, eukaryote cells normally transit to a specific state defined by the realization of its genetic network that specifies the gene expression and protein abundance levels. In the landscape of the cell state space, there exist attractors corresponding to different cell fates [1-4] and barriers between these attractors constrain cells to one attractor (one cell fate). Perturbations such as overexpression of a set of genes may push cells overcome the barriers and thus move from one attractor to another in the cell state space. An example is the generation of induced pluripotent stem cells (iPSCs) from differentiated somatic cells by overexpression of several genes [5-8]. A challenging problem is how to efficiently find effective, ideally the optimal, perturbations to reprogram a cell's fate. In addition, there are other unanswered questions such as how exactly cellular reprogramming occurs and what fundamental principles govern the reprogramming.

We attempt to tackle these challenges from a systems biology point of view and conduct a proof-of-concept study in the model organism budding yeast *Saccharomyces cerevisiae*. Yeast cells proliferate in rich medium through cell cycles and sporulate when nutrients are limited in the environment. We choose these two important biological processes to illustrate how the cell fate can be reprogrammed by perturbing yeast cells, for example, such that they go through cell cycles under sporulation condition or vice versa.

To develop reprogramming recipes and gain mechanistic insights of the reprogramming process, we first assembled a network that regulates cell cycle and sporulation in yeast, which is feasible given the abundant data available. The next steps were to predict phenotypic consequences of perturbations to the cell (yeast cells going through cell cycles or completing sporulation), and to estimate the landscape of the cell state space to monitor the reprogramming process. Methods have been proposed to predict phenotypes but none of them is applicable to search for reprogramming recipes. For example, one group of methods predicted phenotype of knocking out a gene as the phenotypes shared by its neighbor genes in the genetic network [9,10]. This approach does not serve the purpose of finding reprogramming recipe, which requires *de novo *prediction of phenotype. Landscape in the cell state space has also been determined by estimating the probability of each state or solving differential equations to define the dynamics of the system [11,12]. However, these methods are limited to small circuits/networks that cannot provide sufficient molecular details of how reprogramming is achieved.

In this proof-of-concept study, we modeled the genetic network underlying cell cycle and sporulation in yeast using a simple graphical model, Boolean network [13]. We defined the attractors of cell cycle and sporulation using marker genes. Then, using the concept of cell state landscape and a sampling strategy, we illustrated that both quantitative (sporulation efficiency) and qualitative (cell viability upon mutations) phenotypes can be satisfactorily predicted from the network structure without any additional information. Such *de novo *phenotype prediction allows systematic search for perturbations that force the yeast cell to sporulate under the growth condition or vice versa. Once the reprogramming path was defined, the potentials of cell states on the path were estimated by a sampling strategy, which avoids the difficulty of estimating the landscape for all possible cell states but still sheds light on understanding how the reprogramming proceeds from a landscape point of view. Consistent with the landscape concept, we found many perturbations that may serve as reprogramming recipes. More importantly, our analyses suggest that successful reprogramming relies on the heterogeneity of the original cell state (whether a reprogramming can occur) and the restoration of the heterogeneity of the natural cells (how similar the reprogrammed and natural cells are).

## Results

### The network regulating cell cycle and sporulation in budding yeast

**Figure 1:**
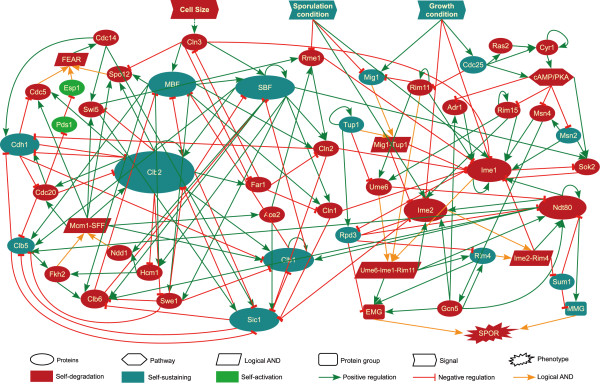
**Genetic network of budding yeast regulating cell cycle and sporulation**. Positive and negative regulations are represented by green arrow and red link with bar, respectively. Orange arrows represent inputs for a logical AND node. Self-degradation, self-sustaining and self-activation nodes are colored in red, blue and green, respectively. See also Additional file 1, Table S1.

Cell cycle and sporulation are two important biological processes that have been well studied in budding yeast. We have curated literature to assemble the key regulators into a 56-node network (Figure 1 and Additional file 1, Table S1), including 44 proteins/complexes (e.g. MBF and SBF), 5 logical AND nodes, two gene groups (i.e. EMG and MMG), one pathway (i.e. cAMP/PKA), one phenotype (i.e. sporulation) and three signal (cell size, sporulation and cell cycle condition) nodes.

Besides the nodes representing proteins, logical AND nodes were used to model the cooperation between multiple proteins that is required for regulating their target genes/proteins. For example, the node of FEAR represents Cdc14 early anaphase release network, which includes proteins such as Esp1, Spo12 and Cdc5 [14], and was thus modeled as an AND node of these proteins. Early and middle meiotic genes are required to be active for sporulation progress; they were collectively represented by EMG and MMG nodes, respectively. Note that the sporulation phenotype is an AND node because it is activated only if both EMG and MMG are on. The cAMP/PKA signaling pathway suppresses the activity of several major sporulation activators such as Rim15 and Msn2, and was represented by one node for simplicity [15]. Since genetic network is dynamically realized in response to environmental cues, we deployed two signal nodes, growth and sporulation conditions, to mimic the medium conditions suitable for cell growth or sporulation induction. The "cell size" node represents the checkpoint entering S phase in the cell cycle if the yeast cell grows to a critical size [16,17].

The curated network represents the current knowledge of cell cycle and sporulation in budding yeast. Under growth condition, when the cell grows large enough to trigger the cell-size signal, Cln3 is activated and it in turn activates MBF and SBF, which induces the transcription of cyclin Clb5 [18]. The activation of Clb5 drives the cell into S phase when DNA replication begins [19,20]. Then the transcription factor complex Mcm1/SFF is formed to activate Clb2, which controls the entry into M phase for segregation [21]. The exit from M phase requires degradation of Clb2 by Cdh1 and Sic1. The cell then goes to the stationary G1 phase before it grows to a critical size and conducts another round of division. Under sporulation condition, the master meiotic regulator Ime1, repressed by growth condition, is turned on [22]. Ime1, Ume6 and Rim11 form a complex to activate early meiotic genes (EMGs) including NDT80 and IME2 [15]. MMG and NDT80 are initially repressed by Sum1 in the vegetative growth condition. Activation of the kinase Ime2 inhibits Sum1's activity, which derepresses NDT80. Ndt80 then transcribes MMG as well as IME1, IME2 and itself to form positive feedbacks. Completing sporulation requires transcription of both EMG and MMG [22].

In the network, we assigned each node to one of three configurations if the incoming activation and repression signals are equal: self-degradation (turned off), self-sustaining (state unchanged) and self-activation (turned on). A self-degradation node tends to be off, which is a simplification of degradation process. For example, Cln1, Cln2 and Cln3 are degraded by a complex SCF associated with Grr1 [23,24]. These degradation mechanisms are not explicitly represented in the network. Self-sustaining nodes denote proteins maintaining a constant level. For example, a self-sustaining protein Cdh1 only functions in the G1 phase but its mRNA level remains constant through the entire cell cycle [25,26]. The separin Esp1 and the securin Pds1 are involved in both meiotic progression and mitotic cell cycle arrest [27-29]. The transcription of ESP1 is constant through the cell cycle [21] and the location of Pds1 is cell-cycle dependent [30,31]. Denoting them as self-activation was to mimic the unknown mechanisms that activate these two proteins.

### Fixed points and dynamics of the network

**Figure 2:**
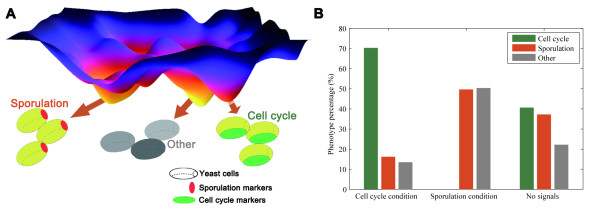
**Network landscape**. (A) Schematic illustration of the network landscape and fixed points of the curated network. Sporulation and cell cycle fixed points are determined by the states of their markers. Fix points other than cell cycle and sporulation are collectively denoted as "Other". (B) Basin sizes (percentage of the 1,000,000 randomly selected initial states converged to the corresponding attractors) of the three attractions given different signals. See also Additional file 2, Figure S2.

The stationary states of the network in Figure 1 reflect cell fates, either completing sporulation or going through cell cycles. To define the stationary states corresponding to sporulation is straightforward: the SPOR node is on, which requires on of both EMG and MMG such that the cell is committed to sporulation [22]. To define the stationary G1 state in cell cycle, under growth condition we first evolved 1,000,000 randomly selected states and find the stationary state with the maximum basin (the largest attractor). We then identified all the proteins/complexes (27 out of 56) in the network showing cyclic change of its state when evolving to the largest attractor. The stationary G1 states of cell cycle were reached if the states of these 27 nodes were the same as those in the largest attractor (the remaining nodes could be in any state). Unsurprisingly, we did observe the known activation of Cdh1 and Sic1 in the stationary G1 state. Note that the temporal evolution of these 27 nodes indeed follows the biological pathway of cell cycle after the cell size signal is turned on (same as that in [16]): from the excited G1 state (the START) to the S phase, the G2 phase, the M phase, and finally back to the stationary G1 state (Additional file 2, Figure S2). This observation validates the representation of cell cycle phases and attractors using these 27 nodes (see Materials and methods). Attractors other than sporulation and cell cycle are collectively referred as "other" (Figure 2A).

Differential equations have been successfully used to study detailed dynamics of yeast cell cycle [32]. Since the genetic network shown in Figure 1 also includes many genes involved in sporulation, most kinetic parameters needed are unknown. In addition, solving differential equations for the network with 56 nodes is difficult if not impossible. Therefore, we exploited Boolean network to identify the fixed points of the network, which should be same as those identified by differential equation based analysis. Since it is impossible to enumerate all the states, we used a sampling strategy to define attractors and basins (see Materials and Methods) which still captured the major events in the cell state space (see discussion in Additional file 2). The two condition signals in our model give three proper combinations since they are mutually exclusive and do not present simultaneously. We first randomly initialized the states without any signal, and follow their evolution to the stationary states. The percentage of the initial states converging to a specific attractor reflects the basin size of the attractor. Without specifying signal, the basin sizes of the three attractors were comparable (Figure 2B). When either cell cycle or sporulation signal is turned on, as expected, the cell prefers the corresponding attractor: under growth condition, 70% and 16% of initial states converged to cell cycle and sporulation attractors, respectively; under sporulation condition, these percentages were <0.1% and 50%. Under either condition, a significant portion of initial states converged to the "other" attractor (neither cell cycle nor sporulation), which illustrates the stochasticity in the network.

Although it is prohibitive to calculate the stable probability of each state for the network of this size [11], the attractors and basins identified by the sampling strategy still paint a global view of the cell state landscape, as conceptually illustrated in Figure 2A. There are three major attractions: sporulation, cell cycle (stationary G1) and other. The yeast cell state is mainly determined by the environmental condition but stochasticity does exist to affect the cell fate. The attractions were robust subject to perturbations to the network structure by adding, deleting or switching direction of edges (Additional file 2, Figure S3), and dynamic trajectories are more convergent in cell cycle than in sporulation (Additional file 2, Figure S4, S5).

### Predicting phenotypes based on genetic network

#### Predicting sporulation efficiency

Effect of deleting a single gene on the efficiency of yeast sporulation has been quantitatively assessed using microarray [33]: a Prespo/Spore ratio for each deletion strain is the ratio between the numbers of cells not-completing and completing sporulation. Such a quantitative phenotype provides a unique opportunity to examine how well network-based predictions can be made *in silico*. We calculated the percentage of 10,000 randomly sampled initial states that converge to sporulation attractor, i.e. an attractor with the SPOR node on, in wild-type and perturbed networks (Materials and Methods). A ratio between sporulation percentage in the wild type and a specific perturbation is computed as the Prespo/Spore ratio and compared with the experimental value.

**Figure 3:**
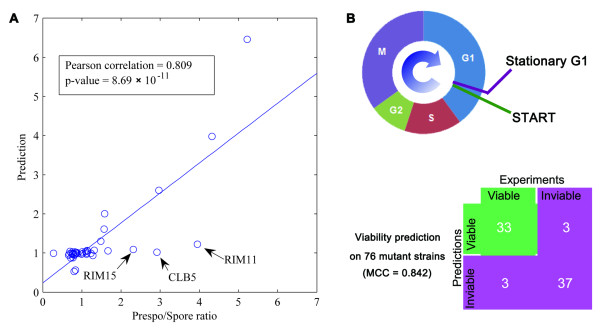
**Predicting phenotypes from the network**. (A) Correlation between the experimental measured (Prespo/Spore ratios) and the predicted sporulation efficiency. (B) Predicting viability of 76 mutants (36 viable and 40 inviable). Strains going through START, G1, S, G2, M, G1 and ending at stationary G1 are predicted as viable. See also Additional file 2, Table S2.

We conducted single gene deletion for all the gene nodes in the network and the corresponding node was clamped to "0" in the simulations. The predicted sporulation efficiencies correlate well with the experimental values, as shown by a Pearson correlation of 0.809 with a P-value of 8.69 × 10^-11 ^given by t-test (Figure 3A) and a Spearman rank correlation of 0.687 with a P-value of 5.08 × 10^-7^. The lower Spearman correlation is because most genes in the network are neither sporulation deficient nor efficient whose Prespo/Spore ratios crowd around 1.0. To further confirm the significance of the correlation, we generated 10,000 random networks, in which the number of nodes, each node's incoming- and outgoing-degrees, the numbers of each node's activators and repressors are same to the curated network (the links to the logical AND and SPOR nodes were not randomized). The correlations between the Prespo/Spore ratios and the predicted sporulation efficiencies based on the random networks only give a mean value of 0.0204 and standard deviation of 0.1835. Compared to the random networks, the predicted correlation of the curated network is extremely significant (P-value = 4.04 × 10^-23^).

This encouraging result suggests that (1) the most prominent interactions related to sporulation have been captured by the curated network even though about half of the nodes mainly function in cell cycle; (2) quantitative phenotypes can be predicted using the cell state landscape concept. There is room for improvement and we did observe three outliers in the predictions: Rim11, Rim15 and Clb5. It is most likely that there are still missing regulatory interactions in the network. The simple Boolean function may not be sufficient to capture complicated regulatory logics either (see discussion in Additional file 2).

#### Predicting viability of mutation strains

We have collected the viability data on 76 mutants from the Saccharomyces Genome Database (SGD [34]) and the study of Chen et al. [32]. To be viable, when started from the stationary G1 state triggered by cell size signal, the yeast cell should go through the cell cycle phases in the proper order. Therefore, in the Boolean network, a viable mutation state must (1) converge to the stationary G1 phase (2) through a trajectory containing all the right-ordered cell cycle phases, under cell growth condition. Out of 76 mutants, 70 (92.1%) predictions match with the literature (Figure 3B and Additional file 2, Table S2), which gives satisfactory classification accuracy (the MCC is 0.842). To evaluate the significance, we predicted the viability of these mutant strains using 10,000 random networks as those in the previous section. All mutations were predicted to be inviable. Although the accuracy was 52.6% (40 out of 76 mutants), the MCC was zero, which suggests that the prediction is just random and insignificant. This satisfactory result shows that the curated network captures the major dynamics of yeast cell cycle and the synchronous updating scheme of Boolean network is appropriate to study the dynamics of this system.

### Reprogramming cell fate

#### Diverse recipes can achieve cellular reprogramming

The satisfactory results of predicting phenotypes based on the curated network encourage us search for perturbations to the network that may reprogram cell fate, e.g. yeast cells sporulate under growth condition or proliferate under sporulation condition. Inspired by the generation of iPS cells [5-8], we searched for perturbation (knockdown or overexpression) combinations of 1 to 4 proteins that can achieve reprogramming. To mimic the generation of iPS cells using transient perturbations, we initialized the perturbed proteins to "1" for overexpression or "0" for knockdown and then let the nodes evolve. There are 1,886,248 possible perturbation combinations for the 42 proteins in the network. Given a condition, we calculated the percentage of the 10,000 randomly sampled initial states converged to the cell cycle or sporulation attractor with and without a perturbation. If a perturbation reverses the percentages of the wild type, reprogramming is achieved and the percentage of the targeted attractor defines the potency of the perturbation. Interestingly, many such reprogramming perturbations (the 100 most potent recipes listed in Additional file 3, Table S3) were found, which indicates the cellular plasticity [4] and is consistent with the epigenetic landscape model that there exist various transition routes between two attractors [1-4]. The potency of reprogramming recipes for sporulation under cell cycle condition is much higher than proliferation under sporulation condition, which indicates the easiness of the reprogramming.

Unsurprisingly, the most potent reprogramming perturbations are combinations of overexpression and knockdown. Intuitively, the most effective way to reprogram cells from fate A to fate B would be repression of genes promoting fate A and overexpression of genes promoting fate B. For example, to reprogram yeast cells to sporulate under the growth condition, the top perturbation is overexpression of GCN5 and knockdown of RPD3, SUM1 and TUP1 that achieves 97% of sporulation. In contrast, perturbations that only contain either overexpression or knockdown have much lower potency of reprogramming: the best overexpression- and knockdown-only perturbations respectively achieve 48.7% (overexpressing IME1, IME2, MSN4 and RIM4) (not in the top 100 recipes) and 85% (knocking down RIM11, RPD3, SUM1 and TUP1) of sporulation under the growth condition. Similarly, all the top 100 recipes reprogramming sporulation to cell cycle under sporulation condition are combinations of overexpression and knockdown.

We next examined which perturbations are most frequently present in the recipes that achieve reprogramming (Table 1 and Additional file 3, Table S3). In both directions of cell fate reprogramming, perturbations to Ime1 and Tup1 have a dominant presence. Ime1 is a major meiosis regulator and it is not unexpected that its overexpression under cell cycle condition or knockdown under sporulation condition helps reprogram the cell fate. Tup1 represses many genes involved in a wide variety of physiological processes [34]. In our model, Tup1 forms a complex with Mig1 and activates Rpd3. Both Mig1 and Rpd3 occur frequently in reprogramming recipes (Table 1). The significance of Rpd3 in reprogramming cell cycle to sporulation is in concert to the observation that Rpd3 is a negative regulator of early meiotic genes and *rpd3 *deletion induces expression of early meiotic genes even in vegetative cells [35]. Since Mig1 is activated by abundant glucose in the environment [36], under the sporulation condition, overexpressing MIG1 mimics the presence of nutrient to drive the cells to grow. Therefore, the importance of Tup1 in reprogramming is likely due to its functional interactions with Rpd3 and Mig1 (Figure 1). Sum1 represses the expression of NDT80 and other middle meiotic genes under growth condition [15,37]. It is not surprising that SUM1 knockdown helps reprogram cells to sporulate under cell cycle condition. Knocking down Msn4, a master regulator of stress response [34], to reprogram yeast cells to proliferate under sporulation condition is also reasonable because Msn4 is repressed by growth condition and it activates Ime1, the master regulator of sporulation.

**Table 1:** Frequencies of the top reprogramming perturbations

A. Reprogramming cells to sporulation under growth condition				
Perturbation	*TUP1*^*KD*^	*RPD3*^*KD*^	*SUM1*^*KD*^	*IME1*^*OE*^

Frequency	0.251	0.223	0.206	0.035

B. Reprogramming cells to cell cycle under sporulation condition				

Perturbation	*MIG1*^*OE*^	*IME1*^*KD*^	*MSN4*^*KD*^	*TUP1 *^*KD*^

Frequency	0.25	0.215	0.138	0.128

*KD*: knockdown; *OE*: overexpression. Perturbations are either gene knockdown or overexpression. See also Additional file 3, Table S3.

#### Cellular reprogramming by perturbation and switch of condition

**Figure 4:**
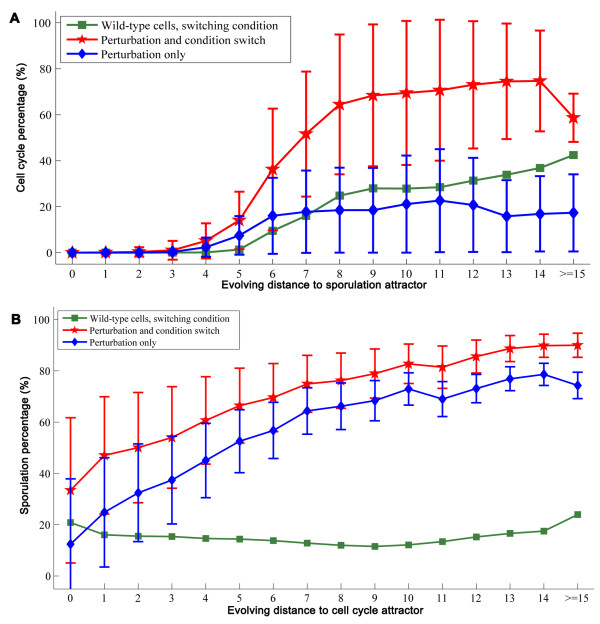
**Dependence of reprogrammability on the heterogeneity of the cell population in the original phenotype**. Means and variations of the most potent 100 recipes are shown. (A) Reprogramming from sporulation to cell cycle. (B) Reprogramming from cell cycle to sporulation.

We examined how the fate of yeast cells can be reprogrammed by both perturbations and signals. First, under sporulation condition, we evolved 10,000 random initial states, and only recorded the initial states and those on their evolving paths converged to the sporulation attractors, i.e. the states in the sporulation basin. We have sampled 3,000 of such basins that include 15,181,093 states in total. Second, upon switching to the growth condition, we found that not all the states obtained in the first step evolved to the cell cycle attractor (the reprogrammed state). The percentage of reprogrammed states among states with the same evolving distance to the sporulation attractors were calculated (Figure 4A). Obviously, the closer to the sporulation attractor (short evolving distance), the less cells were reprogrammed when switching condition from sporulation to growth. Specially, states within 4 evolving steps to the sporulation attractor never evolved to the cell cycle attractor for the wild type cells. It is consistent with the knowledge that yeast cells would complete sporulation once the commitment step is passed, even if they are transferred back to rich medium [22].

We next selected the most potent 100 perturbations that can reprogram cells from sporulation to proliferation under the sporulation condition (see Additional file 2). In addition to each perturbation, we also switched the condition to growth. Combination of perturbation and condition switching mimic the approach of generating iPS or transdifferentiated cells, in which perturbations are introduced to cell type A and the cells are cultured in medium (conditions) for cell type B to reprogram cell type A to B. We grouped cell states based on their evolving distance to the sporulation attractor. The percentage of the states in each group that evolve to the cell cycle attractors for the wild-type and perturbed cells were obtained (Figure 4A). The perturbation does facilitate reprogramming especially from the states that are at least 5 steps away from the sporulation attractors. However, most of the recipes still cannot reprogram cells within two evolving steps to the sporulation attractor (Figure 4A). We conducted the same analysis on the other direction of reprogramming. When condition was switched from growth to sporulation, even the cell cycle attractor states could sporulate because it is a normal sporulation process for the wild-type cells but additional perturbations did boost the sporulation percentage (Figure 4B).

The difference between the two reprogramming directions is biologically meaningful. Yeast cells sporulating under sporulation condition is a natural process and additional perturbations facilitate its progress. In contrast, if yeast cells are committed to sporulation, switching back to proliferation under sporulation condition is cellular reprogramming that requires appropriate perturbations; even with reprogramming perturbations, the fate of yeast cells may not be changed if they are very close to complete sporulation (two evolving steps from the sporulation attractors). Also, for the cell states with the same evolving distance to the sporulation attractors, not all of them can be reprogrammed (Figure 4A). Together, our observations indicate that cellular reprogramming may depend on the heterogeneity of the cell population. If the cells are completely specified in one cell fate, it may not be reprogrammable.

#### Mimicking the generation of iPS cell

**Figure 5:**
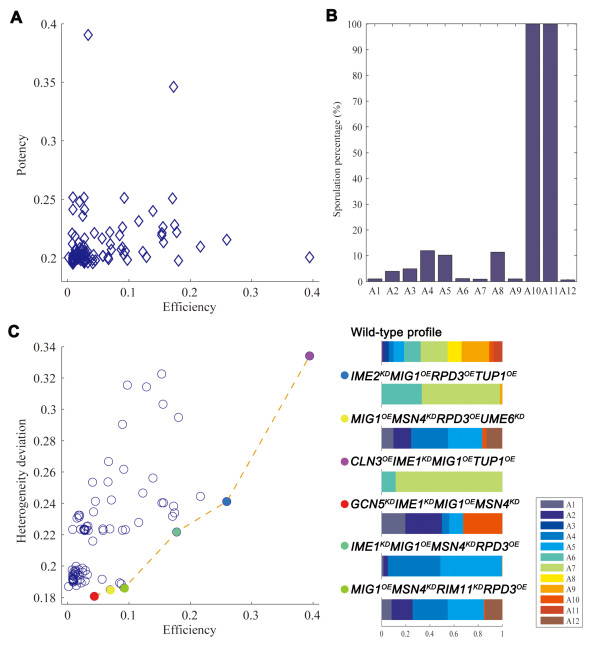
**Divergent properties of recipes reprogramming sporulation to cell cycle under growth condition**. (A) Potency versus efficiency of a recipe. (B) Cell cycle attractors are unequal in terms of their capability to sporulate. When starting from the states within 5 evolving steps to the 12 cell cycle attractors, their percentages of converging to sporulation attractors under sporulation condition vary significantly. (C) Heterogeneity deviation versus efficiency of a recipe. The six Pareto optimal recipes are highlighted on the Pareto frontier. Right panel: heterogeneity profiles for the wild type and reprogrammed cells. Wild type cells under growth condition populate in 12 basins (A1 to A12 and colored differently) and the basin size is proportional to the width of each color in the profile bar. Cells reprogrammed by different recipes show different extent of deviation from the natural cells. See also Additional file 4, Table S4.

It is almost impossible to convert a fully differentiated mammalian cell back to the pluripotent state by only switching culture medium. Therefore, to mimic the generation of iPS cell, we examined the reprogramming of the yeast cells committed to sporulation, i.e. cells within 4 evolving steps from any sporulation attractor that cannot evolve to cell cycle attractors by only switching condition. For the 100 most potent reprogramming recipes, we checked the percentage of the 10,000 randomly sampled cell states that are committed to sporulation but can be reprogrammed to proliferation and we defined this percentage as the efficiency of the reprogramming recipe. Obviously, the efficiency of a high-potency recipe is not necessarily high (Figure 5A). Potency reflects the fixed points of the network under a given environmental condition and reprogramming perturbation, and all initial states are considered when calculating the percentage of cell cycle attractors. Efficiency defined here indicates the convergence to cell cycle attractors only from the cell states committed to sporulation.

Under the growth condition, by random sampling of one million initial states, we found 12 cell cycle attractors and the existence of multiple attractors reflects the heterogeneity of proliferating cells. The reprogrammed cells converge to 2 to 9 of these attractors (Additional file 4, Table S4). Because accumulating evidence suggests that the heterogeneity is critical for the function of embryonic and adult stem cells[38], we hypothesize that the proliferation heterogeneity of yeast cells is also selected by evolution to offer survival advantages. Indeed, the 12 cell cycle attractors and the cell states within 5 evolving steps to them vary significantly on converging to the sporulation attractors when switching condition to sporulation (Figure 5B). This observation indicates that the 12 cell cycle attractors are not equal in terms of their capability to sporulate in response to lack of nutrient in the environment. Therefore, the reprogrammed cells would have different sporulation capabilities depending on which cell cycle attractors are restored.

A deviation metric was defined to measure how much the heterogeneity of natural cells is restored in the reprogrammed cells (Materials and Methods). The more similar the reprogrammed cells populate the cell cycle attractors as the natural cells, the smaller the deviation is. As shown in Figure 5C, an efficient recipe does not necessarily have a small heterogeneity deviation, i.e. it does not restore the heterogeneity of the natural cells well. With respect to the reprogramming efficiency and heterogeneity deviation, five Pareto optimal [39] recipes were identified (Figure 5C). For example, knockdown of IME2 and overexpression of MIG1, RPD3 and TUP1 has a heterogeneity deviation of 0.24 and efficiency of 26%. As discussed above, this perturbation boosts proliferation and inhibits sporulation. The reprogrammed cells repopulate three attractors (the profiles shown in Figure 5C) with similar percentages as the natural cells. This analysis highlights the importance of choosing the optimal reprogramming recipe.

#### Reprogramming routes on the landscape of cell states

**Figure 6:**
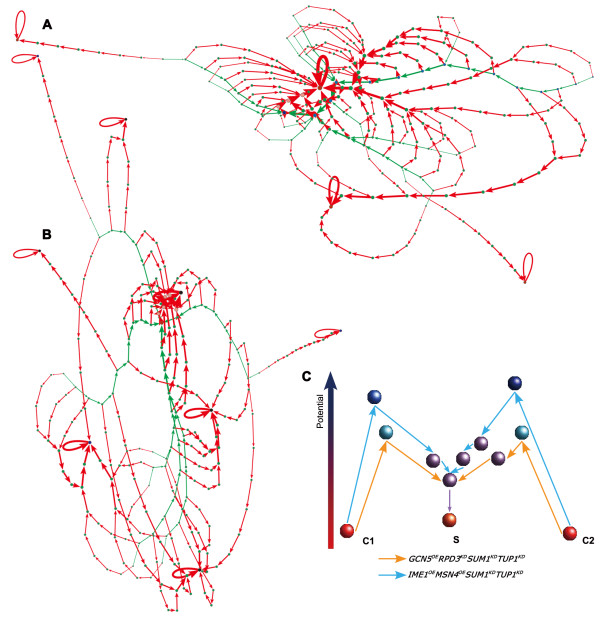
**Monitoring the reprogramming process**. Green and red nodes/links represent wild type and reprogramming paths, respectively. States in the cell cycle biological path are colored in blue. States on the reprogramming path from the wild type attractor to the reprogrammed attractor are colored in pink. Cell cycle, sporulation and other attractors are colored in blue, red and black respectively. The width of the links is proportional to the transition flux. For the clarity of illustration, we only (A) plot 320 transitions between 254 states selected from 9213 transitions between 7191 states in the reprogramming from cell cycle to sporulation under the growth condition by the recipe of *GCN5*^*OE *^*RDP3*^*KD *^*SUM1*^*KD *^*TUP1*^*KD*^, (B) plot 350 transitions between 255 states selected from 14157 transitions between 11089 states in the reprogramming from sporulation to cell cycle under the sporulation condition by the recipe of *IME1*^*KD*^*MIG1*^*OE*^*MSN4*^*KD*^*TUP1*^*OE*^. (C) Example of reprogramming paths from the cell cycle attractors to the sporulation attractors under the growth condition. C1 and C2 are two different cell cycle attractors and S is a sporulation attractor. Each node represents a state on the reprogramming paths and the height reflects the potential in the landscape. See also Additional file 5, Table S5.

To illustrate how the reprogramming proceeds, we monitored the cell state transition for the wild-type and reprogrammed cells. First, under, for example, growth condition we randomly sampled states from the basin of a given attractor state (the root node). Second, we evolved all the states in the previous step under the reprogramming perturbations and added all new states on the evolving paths to this state-transition graph. Figure 6A and 6B show two examples of the state-transitions for both the wild-type and reprogrammed cells. It is obvious that the state-evolving paths are significantly altered by reprogramming perturbations and some nodes act as converging transition states (see below). Exactly as the landscape concept suggests, there are many transition routes between the cell cycle and sporulation attractors.

The common nodes in the reprogramming paths generated by different perturbations were identified transition states in reprogramming. For either reprogramming direction (cell cycle to sporulation or vice versa), the top 100 most potent reprogramming perturbations were used to construct 100 state-transition graphs sampled from 10,000 random initial states. We then calculated a normalized state-transition flux for each state, defined as the flux flowing through the node divided by the number of nodes in the graph. In the 100 state-transition graphs, a transition state was defined as the one that was passed by at least 9,000 reprogramming paths with an average normalized flux larger than 0.05 (more than 5% of the initial states in a state-transition graph passing through these states). With this criterion, no transition state was found in the cellular reprogramming without condition switching, i.e. reprogramming from cell cycle to sporulation under cell cycle condition or from sporulation to cell cycle under sporulation condition. In contrast, transition states did exist for reprogramming with condition switching: three in the reprogramming from cell cycle to sporulation under sporulation condition and five from sporulation to cell cycle under cell cycle condition (Additional file 5, Table S5). The most prominent differences between the transition states of the opposite reprogramming directions are the activities of Mig1 and Rme1: both active in the reprogramming from sporulation to cell cycle but inactive in the opposite direction. As mentioned above, Mig1 is activated by abundant glucose [40] thus promotes mitosis, while Rme1 inhibits meiosis by repressing IME1 expression and promotes mitosis by activating CLN2 expression [34]. Our observation suggests that major transition states may exist even though there are divergent routes of reprogramming generated by diverse reprogramming perturbations.

In the framework of cell state landscape, the reprogramming perturbations pull cells out of one attractor and push it over the barriers to another attractor. To illustrate the landscape, we employed a sampling strategy to estimate the potential of each cell state on the reprogramming paths (Materials and Methods). By defining the reprogramming path and transition states first, we avoided the extensive computation that is infeasible to conduct for a network of this size and devote our efforts to estimate the potential of states only on the reprogramming paths. Figure 6C shows an example of reprogramming paths generated by different perturbations starting from two different cell cycle attractors. It is obvious that the barriers in the landscape are overcome by the perturbations.

## Discussion

### A systematic approach to searching for reprogramming recipes

We propose here a systems biology approach to systematically search for recipes of reprogramming cell fate and understanding its mechanisms. Our approach consists of the following steps. First, constructing a genetic network that regulates the cellular states of interest; Second, appropriately representing the phenotypes using marker proteins/genes; Third, making *de novo *predictions of phenotypes based on the network; Fourth, searching for perturbations to the network that can reverse the phenotype percentages; Fifth, estimating the potential of cell states on the reprogramming paths and find the transition states. In this study of budding yeast, our simulations match well with the experimental measurements and existing knowledge, which indicates the validity of this approach. It is no doubt that improvement can be made in our approach, particularly on constructing a more complete network and predicting phenotypes more precisely. More efficient methods are also needed to estimate potential landscape in the cells state space. Nevertheless, we believe such a framework should be applicable to other systems such as generation of iPS and transdifferentiated cells.

The genetic network with a reasonably large size (56 nodes in this study) can provide mechanistic insights that are often missed by simplified circuits. We have further demonstrated the feasibility of predicting phenotypes from genetic network without additional prior knowledge (Figure 3). This observation is resonant to the proposal that the topology of biological network is dictated by its function [41]. Although we take a simple Boolean network to model the genetic network, it is likely that the network topology encodes the primary constraints of the functional regulations in the cell, which is crucial to *de novo *prediction of phenotypic consequences for perturbations to the system. Tests on additional systems and various phenotypes are needed to further prove this hypothesis.

Our approach allows monitoring of the reprogramming progress (Figure 6). Consistent with the landscape concept, there are many recipes that can achieve a desired reprogramming. We show that recipes with combination of overexpression and knockdown are often more potent than the ones with only either overexpression or knockdown. As discussed above, this observation is quite intuitive but mixed perturbations have not been exploited in generating iPS or transdifferentiated cells, likely due to lack of a systematic method to search for optimal reprogramming recipes. The approach we present here may allow identification of such recipes to effectively reprogram human cells from cell type A to B: knockdown of genes promoting type A and overexpression of genes promoting type B.

We find several transition states (Figure 6) in the reprogramming with condition switching, which is similar to the scenario in the generation of iPS cells. Similar to the checkpoint that yeast cells have to pass to commit to sporulation, mammalian cells often go through several checkpoints along differentiation. It is imaginable that the generation of iPS cells probably needs to overcome several major barriers to achieve pluripotency. This speculation awaits experimental test and, if proved to be true, identification of such transition states would shed lights on understanding the mechanisms of both differentiation and reprogramming.

### Heterogeneity of cell population is crucial for cellular reprogramming

Among the models for iPS cell generation[42], the stochastic model predicts that most cells initiate reprogramming but only a small number complete reprogramming; in contrast, in the elite model only a small portion of cells can initiate and complete reprogramming. Our analyses suggest a compromised model. If cells are fully specified to one cell state as shown in Figure 4, they may not be reprogrammable. Due to the noise inside cell, the cell population showing a common phenotype are heterogeneous; a subset of the cell population that are not completely specified for a phenotypic state can all initiate reprogramming but only a portion of them can complete the desired reprogramming (Figure 4).

### Restoration of heterogeneity of natural cells by reprogrammed cells depends on reprogramming recipe

A critical question in cellular reprogramming is how much the reprogrammed cells resemble the natural cells. The functional roles of heterogeneity of embryonic and adult stem cells are recognized recently; it is believed that the mammalian cells have evolved to achieve such heterogeneity [38] and our observation that the cell cycle attractors are not equal in terms of their capability to sporulate in response to sporulation condition is consistent with this hypothesis. As heterogeneity restoration depends on reprogramming recipes (Figure 5), we argue that an ideal recipe should fully restore such heterogeneity of the natural cells and the observed differences between iPS and embryonic stem cells such as gene expressions may be partially due to the difference of their heterogeneity.

## Conclusions

In summary, our proof-of-concept study on budding yeast illustrates the feasibility of systematic search for optimal reprogramming recipe and highlights the importance of choosing an appropriate recipe to achieve an effective cellular reprogramming. Our analyses also demonstrate that a successful reprogramming may rely on the heterogeneity of the original cell type and suggest a compromised model for generation of iPS or transdifferentiated cells. In addition, restoration of natural cells' heterogeneity by reprogramming is recipe-dependent and we propose such heterogeneity restoration may be critical to the functions of reprogrammed cells.

## Methods

### Boolean network

In a Boolean network [13], the nodes (e.g. genes or proteins) are binary variables and their states can be either on (active "1") or off (inactive "0"). The state of each node is determined by the states of its parent nodes including itself in the case of self-regulation. Network dynamics is modeled by updating the Boolean functions, leading the system transit from the initial state to the final state, where a network state is a collective binary representation of all variables (i.e. a binary vector representing the state of all the proteins).

We adopted a modified version of Boolean network used in [16] and [43], in which the node states in the next time step are determined by the node states of the current time step via the following rules:

where *F *= Σ_*j*_*a*_*ij*_*S*_*j *_(*t*) and *i *is the target node while *j *is the parent node. *S*_*i*_(*t*) is the state of *i *at step *t *and *a*_*ij *_is the contribution weight of parent node *j *to target node *i*. The value of *a*_*ij *_is 1 if *j *activates *i*; or -1 if represses *i*.

### Attractor identification

In principle, all the attractors a dynamic system can be found by enumeration, i.e. evolving the network from all possible initial states and recording the converged states. Because enumeration quickly becomes prohibitive for large networks due to the exponentially increasing number of possible initial states, random sampling strategy is an alternative for attractor identification. It is important to assess the sampling convergence. The sampling was considered converged if the percentages of attractors do not change significantly when increasing sampling. We started from random sampling of 5,000 initial states, and then increased the sampling by 5,000 until the percentages of all the three types of attractors changed less than 1.0%. We found that a random sampling of 100,000 initial states is sufficient to identify all the major attractors of the network.

### Estimating network landscape

To compute the potential landscape of a given Boolean model, Han and Wang generated a transition probability matrix for all the enumerated states in their network of 11 nodes[11]. By solving the master equations, they obtained the steady-state probabilities and thus the potential landscape. For our network of 56 nodes, their method is inapplicable to get the potential landscape of all the possible states. Because we are only interested in states on the reprogramming paths, we employed the following strategy to estimate the potential of the states of interest.

(1) Given input signals, e.g. sporulation condition, we first generated a state-transition graph starting from the states on the reprogramming paths and including the states on their evolving paths to the corresponding attractor;

(2) If the number of states in the state-transition graph was small, we added additional states from randomly sampled evolving paths until the total number of states reached 10,000;

(3) We then calculated the transition probability *T*_*ij *_from state i to state j in the graph using the method of [11]. In our simulation, we set the parameters *μ *= 5 and *c *= 0.001;

(4) Because the state-transition graph was constructed by sampling in step 1 and did not cover all the possible states, we used a pseudo state x to collectively represent all the states that were not included in the graph. The transition probability from a state i to the pseudo state x was calculated as, and the transition probability from the pseudo state x to a state j was, where n is the number of states other than the pseudo state;

(5) We solved the equations using the iterative method of [11] to get the steady-state probability *p*_*i *_for each state;

(6) We repeated step 1 to step 5 to calculate the potential of each state under all three possible conditions, i.e. growth condition, sporulation condition and no signal. We then averaged the steady-state probabilities to calculate the potential of state i as *U *= -ln *p*_*i*_.

### Predicting phenotypes

#### Sporulation efficiency

To estimate the sporulation efficiency, we calculated the percentage of 10,000 randomly sampled states evolving to sporulation attractors. Let it be. To compare the predicted and experimental measured Prespo/Spore ratios determined by microarray for a single gene deletion strain [33], we followed the procedure of [44] to calculate the predicted ratio as

The correlation between the values of and the Prespo/Spore ratios reflects the prediction accuracy of our model.

#### Cell cycle phase and viability prediction

In cell cycle, the "START" checkpoint is responsible for regulating the G1-S phase transition, ensuring that S phase does not begin until the cell reaches the critical size. Although the precise mechanism linking cell size to S phase initiation remains unclear, Cln3 is activated at this checkpoint. Therefore, we modeled this mechanism by a cell-size signal activating Cln3 (Figure 1). There are six phases in a cell cycle: START, G1, S, G2, M and stationary G1 phase. The START phase is different from stationary G1 phase with an active Cln3 node. Cells evolve from START to stationary G1 phase after Cln3 is activated by the cell-size signal.

To determine to which cell cycle phase a state belongs, we identified all the 27 protein nodes that show cyclic state change when converging to the stationary G1 phase and consider them as the indicator of cell cycle phase. For a network state s, we calculate a score for each cell cycle phase *j*,

where *i *is one of the 27 cell cycle nodes, *w*_*ij *_is the weight denoting the contribution of the node *i *to the phase *j *if the node is "on", *s*_*i *_is the activity of node *i *(either 1 or 0). When, *s*_*i *_= 1, the contribution of the node to the score is *w*_*ij*_; if *s*_*i *_= 0, the contribution is 1 - *w*_*ij*_. The network state *s *is considered to be in cell cycle phase *J *that gives the maximum *g(j)*, i.e.

To determine the weight *w*_*ij*_, we started from the biological cell cycle trajectory and initialized the weights of the nodes included in [16] as: *w*_*ij *_= 1 if the node *i *is active in a state belonging to the cell-cycle phase *j*; otherwise *w*_*ij *_= 0. The weights of those nodes not included in [16] are derived from literature. For example, Cdc14 is a protein phosphatase required for mitotic exit. It is localized in the nucleus until transported to cytoplasm in anaphase to decrease the CDK/B-cyclin activity and thus to initiate mitotic exit [45]. Therefore we initially set the weight of Cdc14 in M phase to 1 and 0 in other phases. Next, by manually fine-tuning the weights, e.g. from 1 to 0.8 or from 0 to 0.1, we found the weights that allow correct assignment of each state in the biological cell cycle trajectory to the cell cycle phase (Additional file 6, Table S6). These weights were then used to calculate the *g(j) *score, which can correctly assign network states to the proper cell cycle phases. Take the last state in the biological cell cycle trajectory (Additional file 2, Figure S2) as an example: the scores of START, G1, S, G2, M and stationary G1 are 0.0014, 0.0015, 0, 0, 0 and 0.9971, which correctly assigns the state to the stationary G1 phase.

When judging whether a mutant is viable or not, we clamped the mutated genes (nodes) to 0 (deletion) or 1 (overexpression) and evolved the network from START state of the largest basin under the growth condition. Then we checked (i) whether the obtained trajectory was converged to the cell cycle attractor and (ii) whether the trajectory went through the cell cycle phases in the proper order, namely START - G1 - S - G2 - M - G1 - stationary G1. Note that the G1 phase between M and stationary G1 is optional and the duration of each phase is not necessarily correlated with the number of states involved. If the trajectory proceeds in the proper order of cell cycle phases, the mutant is viable; otherwise it is inviable.

The Matthews correlation coefficient (MCC) measures the quality of binary classifications and is calculated as [46]:

where TP, TN, FP and FN represent true positives, true negatives, false positives and false negatives. A MCC value of +1 represents a perfect prediction, 0 an average random prediction and -1 an inverse prediction.

### Heterogeneity deviation of reprogrammed cells from natural cells

Under growth condition, wild-type yeast cells converged to 12 cell cycle attractor states. The percentages of the random initial states converging to these attractors were denoted by a heterogeneity profile, *w *= (*w*_1_, ···, *w*_*n*_), where *w*_*i *_is the percentage and *n *= 12 is the number of cell cycle attractors. Similarly, the heterogeneity profile for the reprogrammed cells *x *= (*x*_1 _···, *x*_*n*_) was also calculated. The heterogeneity deviation of the reprogrammed cells from the wild-type cells was calculated as

A smaller deviation suggests better restoration of the wild-type heterogeneity by reprogramming.

## Abbreviations

iPSC: induced Pluripotent Stem Cell; EMG: Early Meiotic Gene; ESC: Embryonic Stem Cell; FEAR: Cdc Fourteen Early Anaphase Release; MCC: Matthews Correlation Coefficient; MMG: Middle Meiotic Gene; ODE: Ordinary Differential Equation; SGD: Saccharomyces Genome Database;

## Authors' contributions

SD conceived the entire study, curated the regulatory network, developed the program, performed data analysis and wrote the paper; WW conceived and supervised the entire study, contributed to data analysis and wrote the manuscript.

## Supplementary Material

Additional file 1 **Details of curated regulatory network**. Literature evidence for the nodes and edges in the curated regulatory network. Related to Figure 1 in main text. Click here for file

Additional file 2 **Supporting information**. It describes the robustness and dynamics of the network and more detailed materials related to main text. Click here for file

Additional file 3 **Reprogramming recipes and the frequency of perturbations**. Reprogramming recipes and the frequency of perturbations. Click here for file

Additional file 4 **Recipes reprogramming sporulation to cell cycle**. Recipes reprogramming sporulation to cell cycle. The reprogramming efficiency, heterogeneity deviation, potency and number of restored attractors are listed. Click here for file

Additional file 5 **Common states on the reprogramming paths**. Common states on the reprogramming paths from cell cycle to sporulation under sporulation condition and from sporulation to cell cycle under cell cycle condition. Related to Figure 6 in main text. Click here for file

Additional file 6 **Weights used to assign cell state to cell cycle phase on the biological pathway**. Weights used to assign cell state to cell cycle phase on the biological pathway. Click here for file
