# Supplementary material for: Recipes and mechanisms of cellular reprogramming: a case study on budding yeast Saccharomyces cerevisiae
Source: BMC Syst Biol. 2011 Apr 12;5:50. doi: 10.1186/1752-0509-5-50 (PMC3094211; doi:10.1186/1752-0509-5-50)
Supplement: Additional file 2 — Supporting information. It describes the robustness and dynamics of the network and more detailed materials related to main text. [file 1752-0509-5-50-S2.PDF]

Ding and Wang, Supporting Information

Table of Contents

MAJOR EVENTS ARE CAPTURED IN THE CELL STATE SPACE 1

ROBUSTNESS OF THE CURATED NETWORK 2

DYNAMIC FLOW OF THE NETWORK 2

OUTLIERS OF THE SPORULATION EFFICIENCY PREDICTIONS 3

SUPPORTING TABLES 4

SUPPORTING REFERENCES 7

SUPPORTING FIGURE LEGENDS 8

SUPPORTING FIGURES 9

Major events are captured in the cell state space

The relation between the topology of a regulatory network and the cell state space is very complex. Because we used random sampling in our simulations, it is important to check no major regulatory interaction or cell state is missing. As indicated by the satisfactory performance of Boolean network on predicting sporulation efficiency and cellular viability, we believe that the genetic network we assembled and the Boolean model captured the major topological and dynamic characteristics of the network regulating yeast sporulation and cell cycle. In addition, we also examined the attractors identify by random sampling and enumeration on smaller network with 29 nodes from [1]. We enumerated all the possible $2^{29} = 536{,}870{,}912$ initial states and found 3,320 attractors. We then started from various numbers of randomly sampled initial states and evolved the Boolean network to the attractors. As shown in Figure S1, even a moderate number of 10,000 random sampling found 994 major attractors. To quantitatively indicate the major attractors were correctly identified by random sampling, we calculated the Pearson correlation coefficient of attractor sizes (the number of initial states converged to the attractor) between the random sampling and enumeration. Very significant Pearson correlations were found (>0.99). Theoretical studies have also been conducted to estimate the complexity of the cell state space. For example, Sontag et.al [2] found that the number of limit cycles in the state space is inversely proportional to the number of independent negative feedback loops, while the length of limit cycles in the state space tends to increase as the number of feedback loops increases. However, it is non-trivial to adopt these theoretical methods. Given the satisfactory results of phenotype predictions and comparison between random sampling and enumeration, we believe that no major regulatory relation or cell states are missed.

Robustness of the curated network

To investigate how robust the three attractions are, we perturb the network by edge deletion, addition (adding a positive or negative regulation between unlinked nodes), or switching (changing a positive regulation to a negative one or vice versa). The relative change of each attraction's basin size $\Delta B/B$ [3] is then calculated (Figure S3). For a comparison, we also perform the same perturbations on 2,000 randomly generated networks with each node having the same numbers of incoming- and outgoing-edges as the curated network in Figure 1. It is not surprising that most of the perturbations do not significantly change the basin size, regardless of conditions, phenotypes or perturbation types. This observation is consistent with the previous study on cell cycle [3]. When comparing the curated and random networks, we find slight but consistent differences in all types of perturbations and conditions: basin size change in the curated network is larger for the cell cycle attractor but smaller for the sporulation attractor compared with the random ones. This observation suggests that the yeast network is more resistant to mutations when converging to sporulation, which is probably necessary for the yeast cells to survive the harsh environment that triggers sporulation.

Dynamic flow of the network

To investigate the dynamical stability of the network, we select two representative attractor states for cell cycle and sporulation, and identify all the dynamic trajectories, which form a state-transition graph, from a randomly selected set of initial states under the corresponding conditions (Figure S4). We notice the convergence of the dynamic trajectories to multiple pathways under both conditions. For example, under growth condition, all the dynamic trajectories are converged to the stationary G1 fixed point. Specially, similar to the observation in [3], we also identify the well-known biological cell-cycle pathway and color them in blue in Figure S4. The pathway identified in our study contains more states than that of [3], because our network contains more nodes (56 vs. 11 nodes). Note that the number of states in each phase does not necessarily reflect the actual duration.

To further compare how convergent the dynamic flow to the cell cycle and sporulation attractors are, we define $N(k)$ as the number of states that have a flux k of dynamic flow (the evolution paths of k initial states converged to a state). We find that $N(k)$ decreases as k increases for the two attractors examined above (Figure S5(A)), which can be modeled by power-law with a long tail $N(k) \propto k^{-\gamma}$, where γ is 0.7793 and 0.9346 for the cell cycle and sporulation attractors, respectively (Figure S5(B)). To evaluate whether this difference of γ is statistically significant, we randomly select 3,000 cell cycle or sporulation attractor states, and identify all the dynamic trajectories converging to them

from the random initial states under the corresponding conditions (1,000 runs for each attractor state and each run starting from 10,000 random initial states). We then compare the distributions of the γ for the two attractions. We find that the two distributions are significantly different: means of γ equal to 0.8231 for cell cycle and 0.9427 for sporulation (the p-value for the difference is almost zero). This observation suggests that dynamic trajectories are more convergent in cell cycle than in sporulation. Interestingly, the γ of cell cycle shows a bimodal distribution. To confirm this bimodal distribution is not specific to the attractor state selected, we calculate γ by sampling another 6,923 cell cycle basins and the distribution is still bimodal (Figure S5(C)). This observation suggests that there exist two types of dynamic transitions for cell cycle, but its biological implication and significance wait for further investigation.

Outliers of the sporulation efficiency predictions

We have examined the functions of the three outlier proteins in the sporulation efficiency prediction. Rim11 is a protein kinase required for signal transduction during entry into meiosis. It phosphorylates Ime1 and Ume6 and contributes to the formation of Ime1-Ume6 complex [4]. During cell proliferation, Rim15 is recruited in the nutrient-responsive signal transduction pathway [5]. It is also a known regulator of *IME2* [6]. However, the exact mechanism of Rim11 and Rim15 to promote sporulation is still largely unknown. The third outlier Clb5, a B-type cyclin expressed and accumulated at the G1/S phase in cell cycle, is also involved in meiotic recombination and synaptonemal complex formation [7]. *CLB5* deletion strain indeed fails to sporulate [8], consistent with its large Prespor/Spore value (3.0). Our results indicate that there must be major functional links of Clb5 missing in the network.

Supporting Tables

Table S1. Literature evidence for the nodes and edges in the curated regulatory network. Related to Figure 1 in main text.

Table S2. Predicting the viability of 76 mutant strains. 70 out of the 76 predictions (92.1%) are consistent with the experiments. Related to Figure 3 in main text.

Mutants	Prediction	Experiment	Reference
*ace2*Δ	viable	viable	[9]
*adr1*Δ	viable	viable	[9]
*cdc14*Δ	inviable	inviable	[9]
CDC14	inviable	inviable, G1 arrest	[10]
*cdc20*Δ	inviable	inviable, M arrest	[9]
*cdc20*Δ*, clb5*Δ	inviable	inviable, metaphase arrest	[10]
*cdc20*Δ*, clb5*Δ*, pds1*Δ	viable	viable	[10]
*cdc20*Δ*, pds1*Δ	viable	inviable	[10]
CDC20	inviable	inviable, mitotic catastrophe	[10]
*cdc25*Δ	viable	inviable	[9]
*cdc5*Δ	inviable	inviable	[9]
CDC5	inviable	inviable	[9]
*cdh1*Δ	viable	viable	[10]
*cdh1*Δ*,CLB2*	inviable	inviable	[10]
*cdh1*Δ*,CLB5*	inviable	inviable	[10]
*cdh1*Δ*, cln1*Δ*, cln2*Δ	viable	viable	[10]
*cdh1*Δ*, cln1*Δ*, cln2*Δ*, cln3*Δ	inviable	inviable	[10]
*cdh1*Δ*, cln1*Δ*, cln2*Δ*, SIC1*	inviable	inviable	[10]
*cdh1*Δ*, sic1*Δ	inviable	inviable	[10]
*cdh1*Δ*, swi5*Δ	inviable	inviable	[10]
CDH1	inviable	inviable	[10]
*clb1*Δ	viable	viable	[9]
*clb1*Δ*, clb2*Δ	inviable	inviable, G2 arrest	[10]
CLB1	inviable	inviable, M arrest	[9]
*clb2*Δ	inviable	inviable	[10]
*clb2*Δ*, clb5*Δ	inviable	inviable	[10]
*clb2*Δ*, pds1*Δ	inviable	inviable	[11]
CLB2	inviable	inviable	[9]
*CLB2, cln1*Δ*, cln2*Δ*, cln3*Δ	inviable	inviable, G1 arrest	[10]

CLB2, *sic1*Δ	inviable	inviable, telophase arrest	[10]
CLB2, *swi5*Δ	inviable	inviable	[10]
*clb5*Δ	viable	viable	[10]
*clb5*Δ, *clb6*Δ	viable	viable	[10]
*clb5*Δ, *clb6*Δ, *cln1*Δ, *cln2*Δ	inviable	inviable, G1 arrest	[10]
CLB5	inviable	inviable	[9]
CLB5, *sic1*Δ	inviable	inviable	[10]
*clb6*Δ	viable	viable	[9]
CLB6	inviable	inviable	[9]
*cln1*Δ	viable	viable	[9]
*cln1*Δ, *cln2*Δ	viable	viable, increased cell size	[10]
*cln1*Δ, *cln2*Δ, *cln3*Δ	inviable	inviable, G1 arrest	[10]
*cln1*Δ, *cln2*Δ, *cln3*Δ, *sic1*Δ	inviable	viable, short G1 and very large cell size	[10]
*cln1*Δ, *cln2*Δ, *sic1*Δ	viable	viable	[10]
*cln1*Δ, *cln2*Δ, *SIC1*	inviable	inviable	[10]
CLN1	inviable	inviable	[9]
*cln2*Δ	viable	viable	[9]
CLN2, *cln3*Δ	inviable	inviable	[10]
*cln3*Δ*	inviable	inviable, G1 arrest	[10]
*cln3*Δ, *sic1*Δ	inviable	inviable	[10]
*cyr1*Δ	viable	inviable	[9]
*esp1*Δ	inviable	inviable	[9]
*far1*Δ	viable	viable	[9]
*fkh2*Δ	inviable	viable	[9]
*hcm1*Δ	viable	viable	[9]
*ime1*Δ	viable	viable	[9]
*ime2*Δ	viable	viable	[9]
*mig1*Δ	viable	viable	[9]
*msn2*Δ	viable	viable	[9]
*msn4*Δ	viable	viable	[9]
*ndd1*Δ	inviable	inviable	[9]
*ndt80*Δ	viable	viable	[9]
*pds1*Δ	viable	viable	[10]
PDS1	inviable	inviable	[9]
*ras2*Δ	viable	viable	[9]
*rim11*Δ	viable	viable	[9]
*rim4*Δ	viable	viable	[9]
*rme1*Δ	viable	viable	[9]

*rpd3*Δ	viable	viable	[9]
*sic1*Δ	viable	viable, short G1 phase	[10]
*sok2*Δ	viable	viable	[9]
*sum1*Δ	viable	viable	[9]
*swe1*Δ	viable	viable	[9]
SWE1	inviable	inviable, G2 arrest	[9]
*swi5*Δ	inviable	viable, short G1 phase	[10]
tup1	viable	viable	[9]
ume6	viable	viable	[9]

* Without functional Cdc28, Bck2 shares functions with Cln3 at the G1-S transition[12]. In our model, deletion of *CLN3* also implies the deletion of *BCK2*.

Table S3. Reprogramming recipes and the frequency of perturbations. For each direction of reprogramming (cell cycle to sporulation, or vice versa), top 100 most potent recipes are listed, from which the frequency of each perturbation is calculated. Overexpression is denoted by ":1"; otherwise, knockdown. Related to Table 1 in main text.

Table S4. Recipes reprogramming sporulation to cell cycle. The reprogramming efficiency, heterogeneity deviation, potency and number of restored attractors are listed. Overexpression is denoted by ":1"; otherwise, knockdown. Pareto optimal recipes are highlighted. Related to Figure 5 in main text.

Table S5. Common states on the reprogramming paths from cell cycle to sporulation under sporulation condition and from sporulation to cell cycle under cell cycle condition. Related to Figure 6 in main text.

Table S6. Weights used to assign cell state to cell cycle phase on the biological pathway.

Supporting References

1. Shen L, Chepelev I, Liu J, Wang W: **Prediction of quantitative phenotypes based on genetic networks: a case study in yeast sporulation.** *BMC Systems Biology* 2010, **4:**128.
2. Sontag E, Veliz-Cuba A, Laubenbacher R, Jarrah AS: **The Effect of Negative Feedback Loops on the Dynamics of Boolean Networks.** *Biophysical Journal* 2008, **95:**518-526.
3. Li F, Long T, Lu Y, Ouyang Q, Tang C: **The yeast cell-cycle network is robustly designed.** *PNAS* 2004, **101:**4781-4786.
4. Malathi K, Xiao Y, Mitchell A: **Interaction of yeast repressor-activator protein Ume6p with glycogen synthase kinase 3 homolog Rim11p.** *Mol Cell Biol* 1997, **17:**7230-7236.
5. Cameroni E, Hulo N, Roosen J, Winderickx J, De Virgilio C: **The novel yeast PAS kinase Rim 15 orchestrates G0-associated antioxidant defense mechanisms.** *Cell Cycle* 2004, **3:**462-468.
6. Vidan S, Mitchell AP: **Stimulation of yeast meiotic gene expression by the glucose-repressible protein kinase Rim15p.** *Mol Cell Biol* 1997, **17:**2688-2697.
7. Smith KN, Penkner A, Ohta K, Klein F, Nicolas A: **B-type cyclins CLB5 and CLB6 control the initiation of recombination and synaptonemal complex formation in yeast meiosis.** *Current Biology* 2001, **11:**88 - 97.
8. Dirick L, eacute, on, Goetsch L, Ammerer G, Byers B: **Regulation of Meiotic S Phase by Ime2 and a Clb5,6-Associated Kinase in Saccharomyces cerevisiae.** *Science* 1998, **281:**1854-1857.
9. Dwight S, Harris M, Dolinski K, Ball C, Binkley G, Christie K, Fisk D, Issel-Tarver L, Schroeder M, Sherlock G: **Saccharomyces Genome Database (SGD) provides secondary gene annotation using the Gene Ontology (GO).** *Nucleic Acids Res* 2002, **30:**69-72.
10. Chen KC, Calzone L, Csikasz-Nagy A, Cross FR, Novak B, Tyson JJ: **Integrative Analysis of Cell Cycle Control in Budding Yeast.** *Mol Biol Cell* 2004, **15:**3841-3862.
11. Ross KE, Cohen-Fix O: **The Role of Cdh1p in Maintaining Genomic Stability in Budding Yeast.** *Genetics* 2003, **165:**489-503.
12. Wijnen H, Futcher B: **Genetic Analysis of the Shared Role of CLN3 and BCK2 at the G1-S Transition in Saccharomyces cerevisiae.** *Genetics* 1999, **153:**1131-1143.

Supporting Figure Legends

Figure S1. Attractors identified by random sampling and enumeration on the network from [1]. The number of attractors with different number of random samples and the Pearson correlation of attractor sizes between random samplings and the enumeration are calculated.

Figure S2. Biological cell cycle trajectory. For the clarity of illustration, only the 27 proteins/complex that show periodic state changes are included in the figure. White or black represents protein state “on” or “off”. Cln3 in the START phase of cell cycle is activated by the cell size signal. Related to Figure 2.

Figure S3. Network robustness. The results are calculated from the curated network and 2,000 randomly generated networks under different conditions. The relative changes (percentage) of the basin size for the three phenotypes subject to network perturbations by (A) edge addition; (B) edge deletion; (C) edge switching.

Figure S4. Dynamic trajectories of states converging to (A) cell cycle and (B) sporulation attractors under the growth and sporulation conditions, respectively. Each node is a state. An arrow between two states indicates the dynamic flow. The size and color of a node and the width of an arrow are proportional to the logarithm of the flux flowing through them. The biological cell-cycle pathway converging to the stationary G1 fixed point (red node) is colored in blue.

Figure S5. Dynamic flow of the network. (A) The distributions of number of states $N(k)$ relative to the flux of dynamic flow k when converging to the cell cycle (blue circles) and sporulation (red triangles) attractors. Both distributions obey power-law, $N(k) \propto k^{-\gamma}$ with different γ values. (B) Distribution of γ values. Mean and standard deviation for cell cycle are 0.8231 and 0.0466, respectively. Mean and standard deviation for sporulation are 0.9427 and 0.0219, respectively. (C) Further confirmation of the bimodal distribution of γ values for the cell cycle state transition graphs. Mean and standard deviation are 0.8229 and 0.0466, respectively.

Supporting Figures

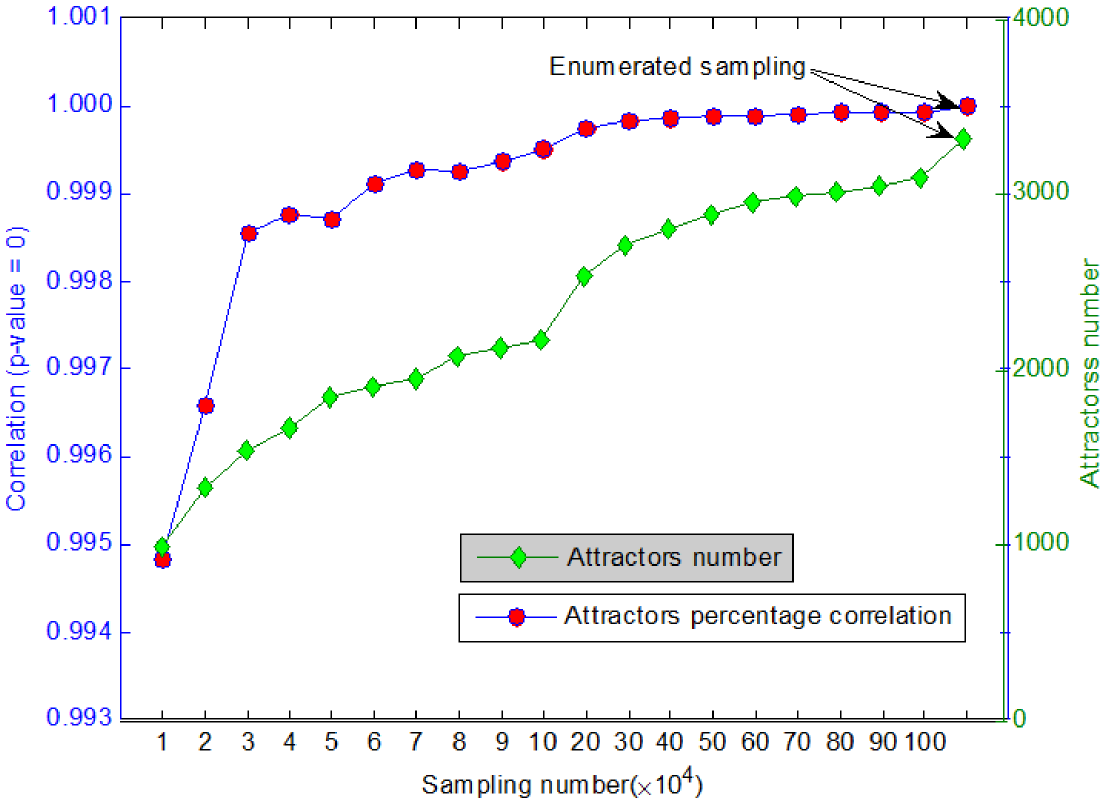

Figure S1

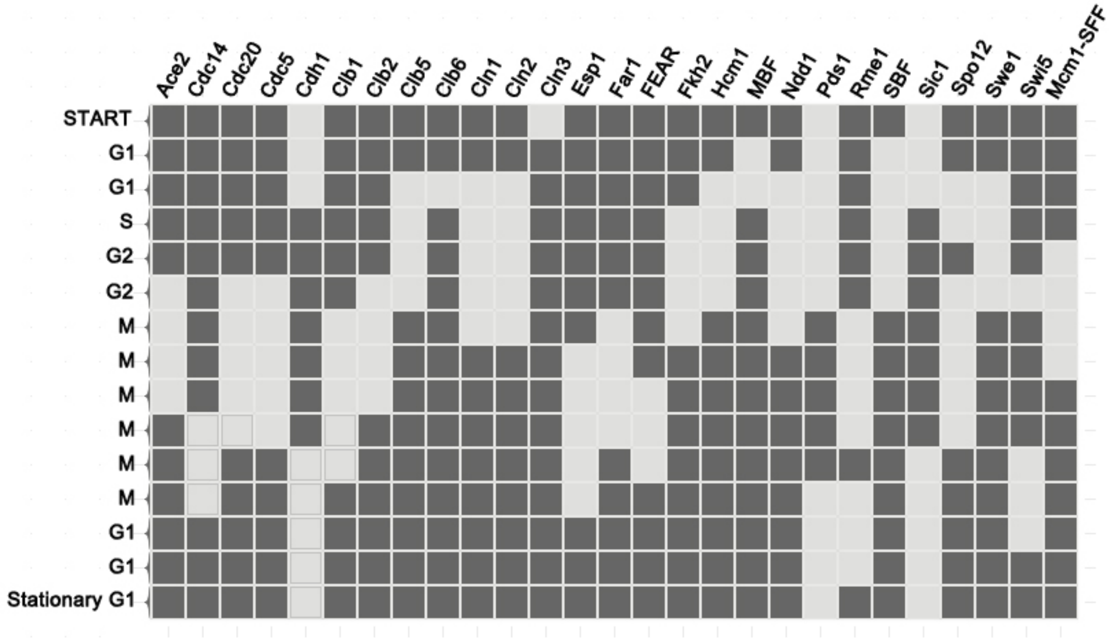
Ace2
Cdc14
Cdc20
Cdc5
Cdh1
Clb1
Clb2
Clb5
Clb6
Cln1
Cln2
Cln3
Esp1
Far1
FEAR
Fkh2
Hcm1
MBF
Ndd1
Pds1
Rme1
SBF
Sic1
Spo12
Swe1
Swi5
Mcm1-SFF
START
G1
G1
S
G2
G2
M
M
M
M
M
M
G1
G1
Stationary G1

Figure S2

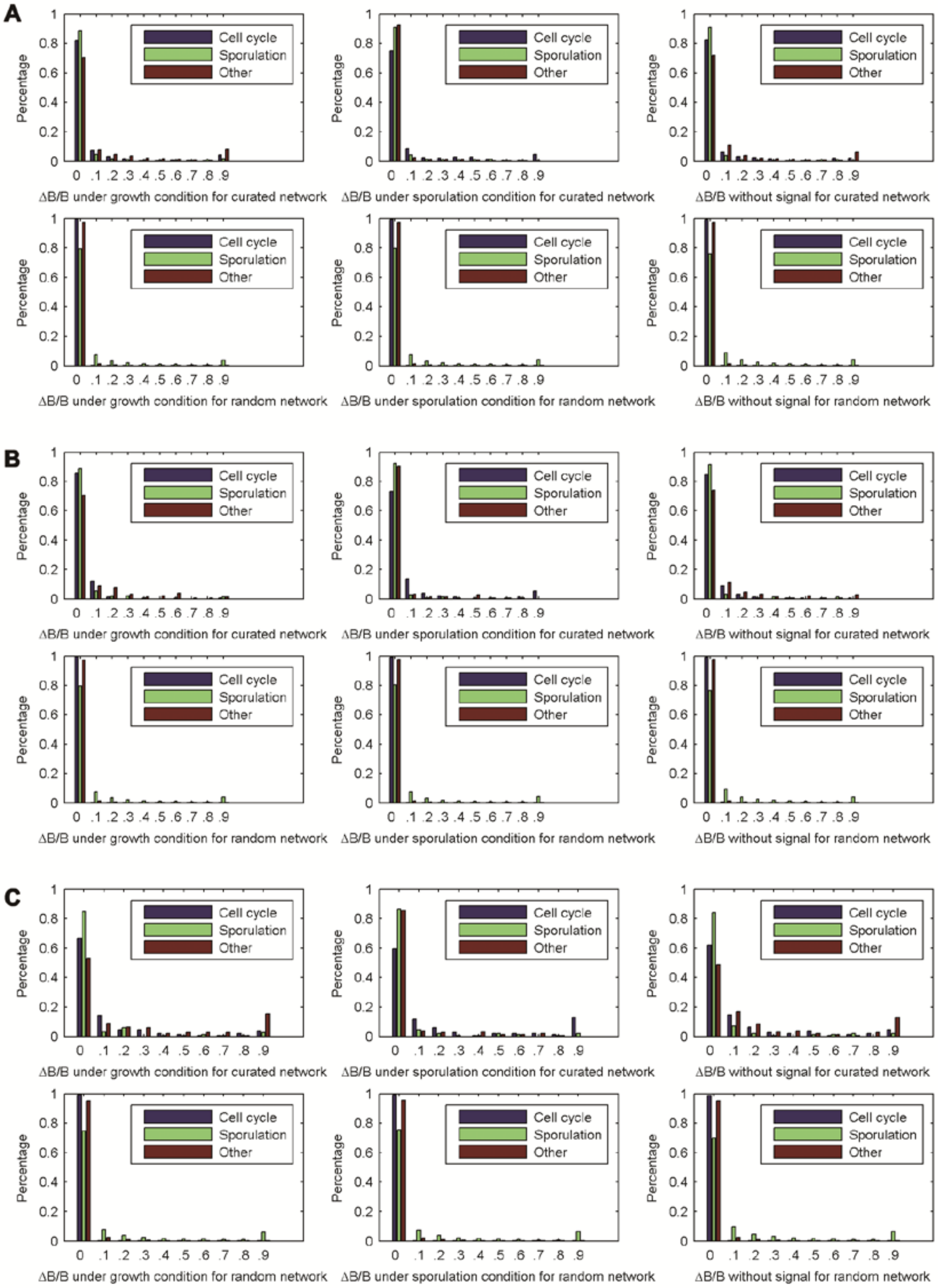
A
B
C
Percentage
Cell cycle
Sporulation
Other
0 .1 .2 .3 .4 .5 .6 .7 .8 .9
ΔB/B under growth condition for curated network
ΔB/B under sporulation condition for curated network
ΔB/B without signal for curated network
ΔB/B under growth condition for random network
ΔB/B under sporulation condition for random network
ΔB/B without signal for random network

Figure S3

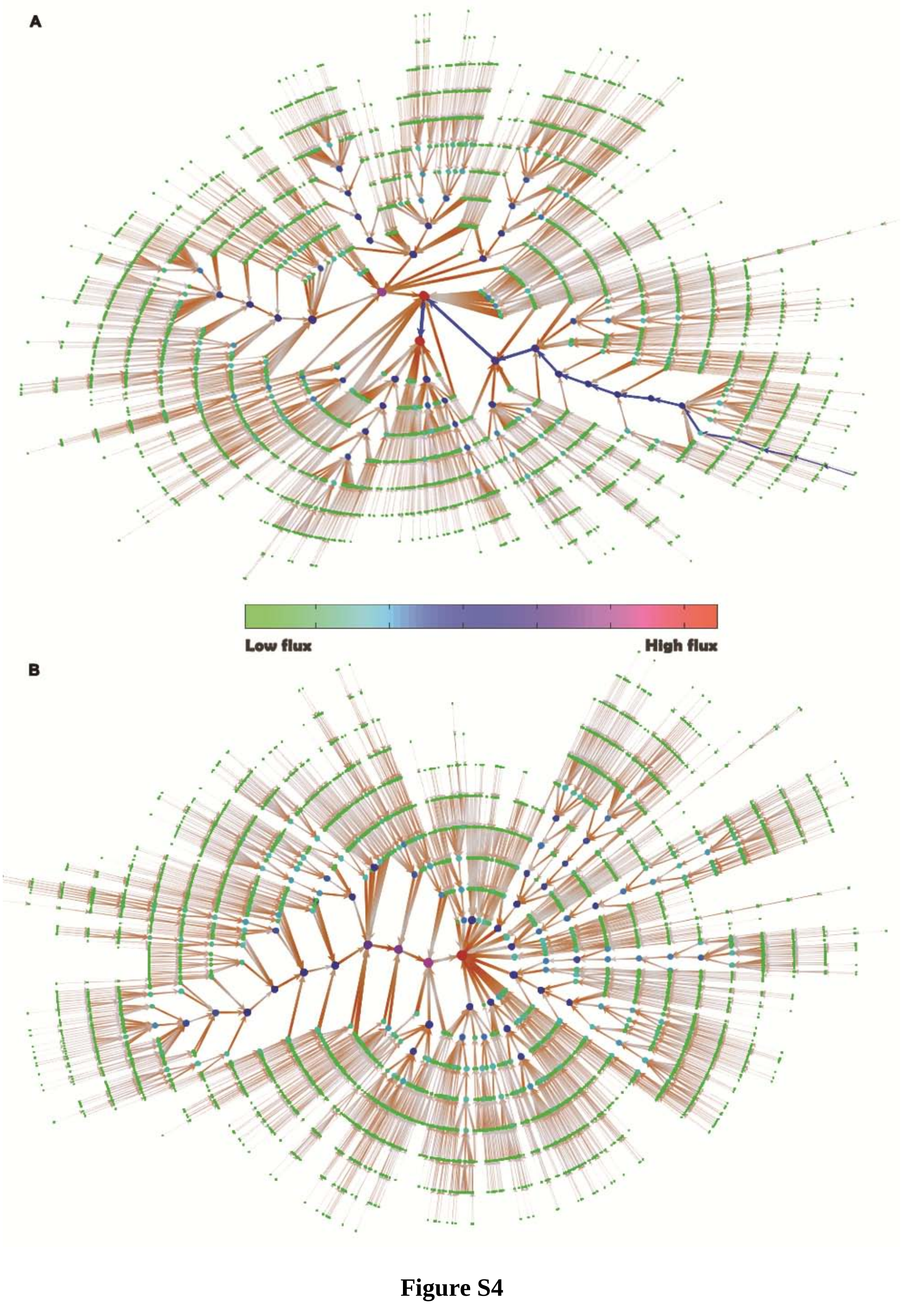

Figure S4

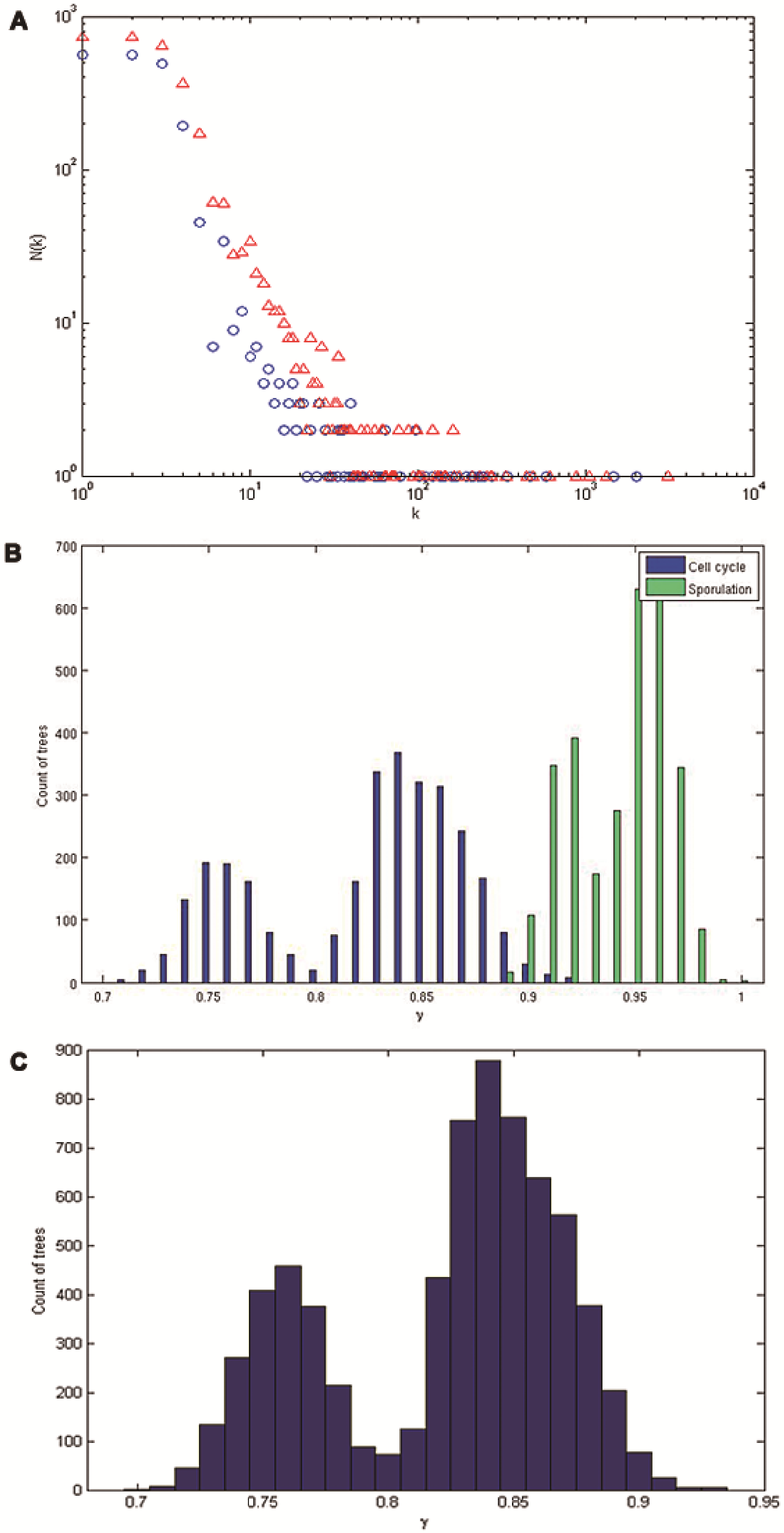
A
N(k)
k
B
Cell cycle
Sporulation
Count of trees
γ
C
Count of trees
γ

Figure S5
